# Designer Drugs: A Synthetic Catastrophe

**DOI:** 10.17756/jrds.2015-014

**Published:** 2015-08-10

**Authors:** James Fratantonio, Lawrence Andrade, Marcelo Febo

**Affiliations:** 1 Division of Applied Clinical Research, Dominion Diagnostics, LLC, North Kingstown, RI, USA; 2 Division of Research and Development, Dominion Diagnostics, LLC, North Kingstown, RI, USA; 3 Department of Psychiatry and McKnight Brain Institute, University of Florida College of Medicine, Gainesville, FL, USA

**Keywords:** Designer drugs, Mephedrone, Methylone, MDPV, α-PVP, Bath salts

## Abstract

Synthetic stimulants can cause hallucinations, aggressive behaviors, death and are sometimes legal. These substances are sold as plant food and bath salts that are “Not for Human Consumption”, therefore skirting the 1986 Federal Analogue Act and giving a false pretense of safety. Studies have proved that these substances are toxic, have a high abuse potential, and are becoming extremely prevalent in the United States. This creates a dilemma for law enforcement agents, hospitals, and substance use disorder treatment centers. Urine Drug Testing is utilized as a clinical diagnostic tool in substance use disorder treatment centers, and the furious pace at which new synthetic stimulants are introduced to the black market are making the detection via urine increasingly difficult. This article will discuss the prevalence, pharmacology and difficulty developing laboratory assays to detect synthetic stimulants.

## Introduction

The Federal Analogue Act of 1986 states:

“A controlled substance analogue shall, to the extent intended for human consumption, be treated, for the purposes of any Federal law as a controlled substance in schedule I.”

Clandestine chemists found a loophole in this language and have for the past decade been able to produce new “legal” designer drugs that are sweeping the nation with popularity, injury and abuse. Synthetic stimulants are “legally” sold under the false pretense as *bath salts,* and *plant food*, and are commonly added to pre-workout supplements [1]. The United States responded to the public health risks by banning the sale, possession, and use of specific designer drugs in the Synthetic Drug Abuse and Prevention Act of 2012. Clandestine chemists have responded to legislation by creating new alternative substances that are more dangerous in an effort to exploit loopholes in legislation to make a legal profit.

Synthetic cathinones are derived from a flowering plant, *Catha edulis*, commonly known as khat. Found in the Arabian Peninsula and East Africa, Khat has been used for centuries as part of established cultural traditions. Chewing the leaves of the plant induces a state of euphoria and increased alertness [2]. Cathinone is the main psychoactive ingredient in khat that clandestine chemists have exploited to synthesize dangerous, addictive synthetic drugs that are being marketed legally to populations all across the world. The intoxicating effects of bath salts include hallucination, psychosis, tachycardia, hypertension, hyperthermia, agitation and violent behaviors.

Drug overdose deaths have become the leading cause of death by injury in the United States, surpassing car accidents. According to the American Association of Poison Control Centers (AAPCC) from January 1, 2011 through December 31, 2014 there have been 10,403 human exposures to synthetic cathinones reported [3, 4]. According to the Drug Abuse Warning Network (DAWN) synthetic cathinones were involved in over 22,904 emergency department visits and 52% of those cases involved synthetic cathinones in combination with other drugs [5].

“Bath salts”, as synthetic cathinones are commonly referred, are dangerous drugs that are not to be confused with Epsom salts, which are added to bath water. Bath salts are sold online under creative marketing names such as Super Coke, Cloud Nine, Ivory Wave and Bliss. Meyers et al. were able to find 31 unique retail websites that were registered in the United States, Germany and the United Kingdom [6]. Schneir et al. analyzed the contents of thirty-five bath salt products purchased from retail stores in California and internet sites located in the United States and the most common synthetic cathinone identified was 3,4-methylenedioxypyrovalerone (MDPV) [7]. They reported that most products contained multiple cathinones and in some cases there were dramatic differences in total cathinone content between products with the same declared weight and even between identically named products. Not knowing what drugs are being taken or at what dosages increases the risk of overdose, adverse reactions, and drug-drug interactions for the user as well as creating a difficult situation for emergency room physicians to treat ill patients appropriately. As Stiles et al. have reported an increased difficulty in treating bath salt-induced psychosis only reiterates that healthcare providers, police, and hospital security personnel must be educated and working collaboratively to provide the best care for these patients [8].

Determining treatment guidelines for bath salt related hospitalizations proves to be difficult and costly, but understanding the pharmacology, laboratory analysis, and legality of bath salts will increase our treatment outcomes and produce standardized treatment guidelines [9, 10]. Current research on the more prevalent bath salts including MDPV, Methylone, α-PVP, and Mephedrone have revealed increased abuse liability, pharmacokinetic metabolism profiles, pharmacodynamic effects, neurotoxicity, and urine laboratory assay development which will be discussed in detail [11-19].

This paper reviews the pharmacology of the most prevalent synthetic stimulants, legality, and the constant battle of trying to detect them in Urine Drug Testing (UDT).

## Pharmacology

**Figure 1:**
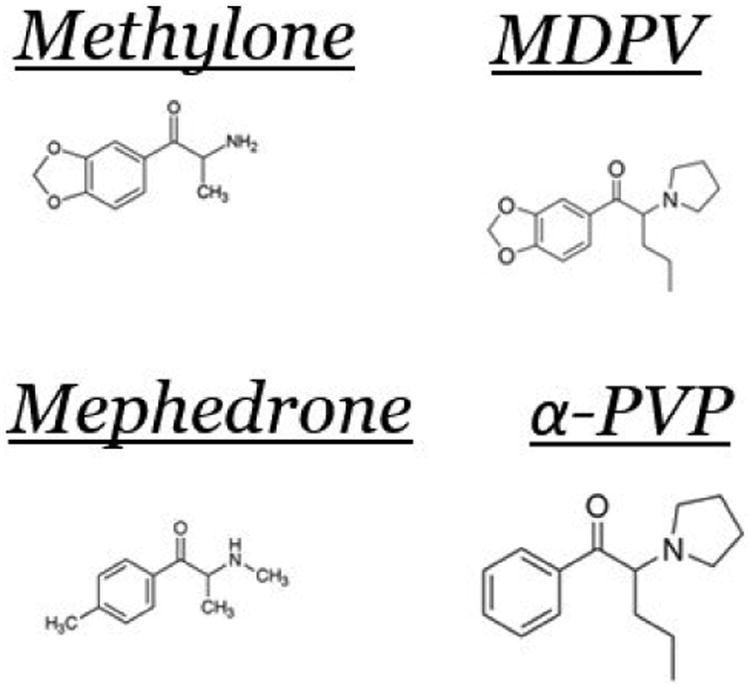
Chemical structures of Methylone, MDPV, Mephedrone and α -PVP.

Synthetic cathinones are phenylalkylamine derivatives that commonly share a beta-ketone moiety and are commonly referred to as “bk-amphetamines” [20] (see figure 1).

The pharmacology of all the synthetic cathinones is not entirely known, but Simmler et al. have determined that they can inhibit the transport of noradrenaline, serotonin, and dopamine; and monoamine receptor binding affinity [21]. Methylone and mephedrone are more similar to 3,4-methylenedioxymethamphetamine (MDMA) by being nonselective transporter substrates that increase the release of dopamine, norepinephrine, and serotonin [22]. MDPV mimics cocaine, and is a transporter blocker that potently inhibits the uptake of dopamine and to a lesser extent norepinephrine. Its effects mediated through powerful effects on dopamine transporter function are thought to mediate its powerfully rewarding as well as its negative after effects [23].

Laboratory rodents have been shown to readily self-administer several of the more popular synthetic stimulants, therefore demonstrating the high abuse and addictive properties of these chemicals [15]. Anneken et al. have discovered that methylone and mephedrone alone do not damage dopamine nerve endings, but when used in combination with methamphetamine they accentuate the neurotoxicity effects [11]. Creehan et al. discovered that the abuse liability of mephedrone, methylone and MDMA are predicted to be similar in female Wistar rats that were trained to self-administer mephedrone, methylone or MDMA, but the liability may be worse if the female rats were initiated on mephedrone [12]. Watterson et al. also revealed that methylone has a reinforcing effect in rats during an intravenous self-administration (IVSA) study through spontaneous acquisition procedures [24]. Furthermore, demonstrating the potential damaging and addictive properties of both methylone and mephedrone.

MDPV was first evaluated for the treatment of chronic fatigue in 1969, but the drug development process was stopped early because of adverse effects including agitation, paranoia, tachycardia and even death [13, 25]. The mechanism of action for the psychostimulant effects of MDPV is unknown, but Baumann et al. have demonstrated that MDPV is similar to cocaine [22]. The *in vitro* test demonstrated that MDPV is a potent monoamine transporter blocker that is selective for norepinpehrine and dopamine transporters, with negligible serotonin transporter activity [13]. Anneken et al. found that MDPV is a non-substrate blocker of the dopamine transporter and protects against methamphetamine neurotoxicity [11]. Anizen et al. discovered that at lower doses MDPV experiences linear pharmacokinetics unlike both methylone and MDMA [13]. Linear pharmacokinetics are easier to dose and have a predictable dose-toxicity profile. MDPV repeatedly is found in blood and urine in fatal cases of bath salt overdoses in the United States [24, 26]. Furthermore, suggesting that MDPV is primarily responsible for the fatal adverse effects of bath salts, possibly because of the pyrrolidine ring and the tertiary amino group that lead to a more lipophilic compound that more easily crosses the blood brain barrier [27]. Febo et al. imaged rats after administration of a single dose of MDPV and discovered a highly significant reduction in functional connectivity across 33 brain regions. Brain functional connectivity has been reported in patients suffering from psychosis and has been linked with cognitive dysfunction, hallucinations, and negative affective states [28]. Moreover, Watterson et al. discovered that MDPV has reinforcing activity, an escalated intake over time, and decreased thresholds for intracranial self-stimulation (ICSS) in rats that would be indicative of a strong potential for compulsive use, addiction, and reward deficiency syndrome in humans [29]. In addition to its powerful rewarding effects in comparison to cocaine, MDPV is reported to produce aversive long duration after effects in tests for conditioned taste aversion [30]. Rats are induced to acquire conditioned taste aversion to saccharine solution following single administration of MDPV doses within the range of 1-3 mg kg^-1^. This effect in rats is consistent with the late onset negative symptoms following MDPV intake that have been reported in the clinical literature of and which adversely impact the mental health of users of this lethal designer drug [31, 32].

As the more prevalent synthetic cathinones were permanently declared illegal by the Synthetic Drug Abuse and Prevention Act of 2012, chemists were developing new synthetic stimulants to stay a step ahead of legislation including the development of alpha-Pyrrolidinopentiophenone better known as α-PVP, or “flakka.” Asarde et al. demonstrated that α-PVP has similar potency to MDPV by training male Wistar rats in intravenous self-administration of α-PVP or MDPV [33]. Considering their relative potency and efficacy, it is hypothesized that MDPV and α-PVP have similar abuse liability. There have been several reports of patients hallucinating, and developing aggressive outbursts while taking α-PVP, and several deaths [34]. The recent popularity of α-PVP in Florida has forced the Attorney General to issue a press release warning parents about the dangers of the drug. According to Florida DEA agents, seizure of α-PVP has tripled in the first half of 2015 compared to the entire year of 2014. Chemists removed the methylenedioxy group from the benzene ring on the MDPV structure to cleverly develop a new substance that is legal if marketed not for human consumption according to the Synthetic Drug Abuse and Prevention Act of 2012.

Chemists have also introduced synthetic stimulants into the dietary supplement market. A year after the DEA identified β-methylphenylethylamine (BMPEA), a stereoisomer of amphetamine, in 21 dietary supplements claiming to contain *Acacia rigidula*, Cohen et al. reported that more than half still contained BMPEA. BMPEA was originally synthesized in the 1930s, but safety and efficacy studies were never performed in humans, and it has remained a research chemical [35]. N,α-diethyl-phenylethylamine (N,α-DEPEA), a methamphetamine analog, was also identified in workout supplements [36]. Ingesting supplements that are believed to be safe that contain synthetic stimulants is a major public health safety concern, can increase the risk for relapse, and introduces unknowing people to addictive substances.

## Lab Identification

Clinicians treating patients with a substance use disorder that present with abnormal behaviors including acute neuropsychiatric complaints should consider adding a designer drug test to the patients UDT. Acute toxicity can be deadly and appropriate intervention is crucial to help aid patients into recovery.

The gold standard of highly selective and specific concentration determination and confirmation of drugs and metabolites in biological matrices in the clinical laboratory is the bioanalytical LC-MS/MS (liquid chromatography coupled with tandem mass spectrometry) assay. For most laboratories these are in-house developed assays. (Laboratory developed tests, LDT's). There are some specific challenges in assay development with respect to designer drugs.

Two main LC-MS methodology approaches can be used for qualitative screening and quantitation of designer drugs. High Resolution Mass Spectrometry/Accurate Mass (HRMS/AM) is the gold standard for performing non-targeted analysis. This type of approach is useful when no chemical structure or reference standard for the designer drug is available. This is an increasingly powerful tool as synthesis of designer drugs is trending to a de novo approach rather than analogues of existing drugs to circumvent laws covering these chemically similar versions. The sample analysis data is collected and stored then re-interrogated at a later date when more information about the suspect drug is known. This type of analysis gives very high confidence results and is used primarily by non-clinical or toxicology laboratories for qualitative analysis. In targeted analysis the aim is to match the data collected from the specimen analysis to a reference standard. This approach uses both HRMS/AM and traditional triple quadrupole analyzers (LC-MS/MS) by clinical laboratories producing quantitative results.

As previously discussed, targeted analysis generally requires the use of a reference standard. Although not specifically required, a certified reference material (CRM) is preferred when developing LC-MS/MS assays. The CRM is selected as opposed to a raw material or in-lab synthesized material as the drug standard must be fully characterized to ensure identification. In addition exact purity has to be established to curtail lot-to-lot variation which would result in inconsistent, inaccurate concentration determination. Because designer drugs evolve so rapidly, the availability of CRM's can have a significant lag time. Commercial suppliers need time to research, synthesize, scale up, and fully characterize these drugs, and sometimes do not target these drug standards until they are scheduled by the DEA. This can result in significant delays in method development and therefore difficult to keep pace with the rapid production of synthetics.

Another challenge is there is little to no pharmacokinetic data available which is used to select the concentration reference range to best interpret data. In addition the parent drug may not be the most informative target as drug metabolites may be higher in concentration or provide a longer detection window (and again the CRM must be available).

## Legality

After increased injury from these designer drugs President Obama signed the Synthetic Drug Abuse and Prevention Act of 2012 that illegalized methylone, mephedrone, MDPV and nine phenylethylamines. This act spurned a need for clandestine labs to design new synthetic cathinones to be marketed and sold as “plant food” to continue legal sales into the United States. Unfortunately, these new synthetic cathinones are being developed and sold quicker than specialty laboratories can develop assays to detect them [6, 19, 22, 36-39]. It takes too long to develop legislation for outlawing new synthetic drugs. Once the Synthetic Drug Abuse and Prevention Act of 2012 outlawed 12 synthetic stimulants, the black market developed new substances. This forced the Deputy Administrator of the Drug Enforcement Administration to temporarily schedule 10 synthetic cathinones into schedule I in March of 2014, which includes α-PVP [40].

## Conclusion

Synthetic stimulants have been repeatedly shown to have addictive properties and promote reward deficiency syndrome and they can cause hallucinations, aggressive behaviors, and death. The government has been behind the eight ball with placing specific stimulants in the category of schedule I, and therefore illegal. This creates a dilemma for law enforcement agents, hospitals, and substance use disorder treatment centers. The furious pace at which new synthetic stimulants are introduced to the black market are making the detection via urine increasingly difficult because of the inability to create CRMs and the lack of pharmacokinetic evidence of the new substances. Having highly addictive substances being sold under the false pretense of being safe has increased the prevalence and incidence of synthetic stimulant poisoning, which has led many to addiction, and even some to death.
